# Targeting the complement system in pancreatic cancer drug resistance: a novel therapeutic approach

**DOI:** 10.20517/cdr.2021.150

**Published:** 2022-04-03

**Authors:** Naushair Hussain, Deea Das, Atreyi Pramanik, Manoj K Pandey, Vivek Joshi, Kartick C. Pramanik

**Affiliations:** ^1^Department of Biomedical Sciences, Kentucky College of Osteopathic Medicine, University of Pikeville, Pikeville, KY 41501, USA.; ^2^Department of Education, South College, Knoxville, TN 37902, USA.; ^3^Department of Biomedical Sciences, Cooper Medical School of Rowan University, Camden, NJ 08103, USA.; ^4^Department of Biochemistry and Molecular Biology, Drexel University College of Medicine, Wyomissing, PA 19610, USA.

**Keywords:** Pancreatic cancer, complement system, immunotherapy, drug resistance, nuclear factor kappa B (NF-κB), signal transducer and activator of transcription (STAT3), c-mesenchymal-epithelial transition factor (C-MET), phosphoinositide-3-kinase/protein kinase B (PI3K/AKT)

## Abstract

Pancreatic cancer is ranked as the fourth leading cause of cancer-related mortality and is predicted to become the second leading cause of cancer-related death by 2030. The cause of this high mortality rate is due to pancreatic ductal adenocarcinoma’s rapid progression and metastasis, and development of drug resistance. Today, cancer immunotherapy is becoming a strong candidate to not only treat various cancers but also to combat against chemoresistance. Studies have suggested that complement system pathways play an important role in cancer progression and chemoresistance, especially in pancreatic cancer. A recent report also suggested that several signaling pathways play an important role in causing chemoresistance in pancreatic cancer, major ones including nuclear factor kappa B, signal transducer and activator of transcription 3, c-mesenchymal-epithelial transition factor, and phosphoinositide-3-kinase/protein kinase B. In addition, it has also been proven that the complement system has a very active role in establishing the tumor microenvironment, which would aid in promoting tumorigenesis, progression, metastasis, and recurrence. Interestingly, it has been shown that the downstream products of the complement system directly upregulate inflammatory mediators, which in turn activate these chemo-resistant pathways. Therefore, targeting complement pathways could be an innovative approach to combat against pancreatic cancer drugs resistance. In this review, we have discussed the role of complement system pathways in pancreatic cancer drug resistance and a special focus on the complement as a therapeutic target in pancreatic cancer.

## INTRODUCTION 

Pancreatic cancer is ranked as the fourth leading cause of cancer-related mortality and is predicted to become the second leading cause of cancer-related death by 2030^[1]^. About 90% of these tumors are pancreatic ductal adenocarcinomas (PDACs), tumors of the exocrine part of the pancreas. The cause of this high mortality rate is due to PDAC’s rapid progression and metastasis, low rate of early detection, and development of drug resistance^[1]^. 

In the last ten years, gemcitabine has been the most common treatment option for advanced-stage pancreatic cancer. However, even with this treatment option, the 5-year survival rate is still incredibly low, only 10% according to the American Cancer Society^[2]^. Unfortunately, the survival rate is continuing to decrease due to increased incidences of chemoresistance in pancreatic cancer cells. Researchers have investigated to determine the mechanism of chemoresistance to improve the efficacy of gemcitabine and other chemotherapeutic drugs. It has been found that a multitude of signaling pathways play a role in causing chemoresistance, the major ones being nuclear factor kappa B (NF-κB), signal transducer and activator of transcription (STAT3), c-mesenchymal-epithelial transition factor (C-MET), and phosphoinositide-3-kinase/protein kinase B (PI3K/AKT)^[1,3,4]^. 

Researchers have investigated these different signaling pathways to find a target they could inhibit to prevent their activation and sensitize the cancer cells to the treatment. However, with multiple pathways and a surplus of stimuli causing the activation, it has proven difficult to successfully inhibit them all. It has been found that inflammation which is a known element of cancer progression, plays a significant role in the activation of these different signaling pathways^[5]^.

A huge mediator of inflammation within cancer is the complement system which is a part of the body’s innate immune response and has been shown to induce a series of inflammatory responses to help fight infection and other diseases^[6,7]^. It has been proven that the complement system has a very active role within cancer progression, especially within establishing the tumor microenvironment^[8]^. In addition, it has been shown that the downstream products of the complement system directly upregulate inflammatory mediators, which in turn activate these chemo-resistant pathways^[7]^.

This review article summarizes recent advances related to the role of the complement system in pancreatic cancer chemo-resistant signaling pathways, and the opportunity of targeting the complement system as a novel treatment to overcome drug resistance in pancreatic cancer.

### Overview of the complement system

The complement system is an important mechanism that links innate immunity to adaptive immunity and helps the body fight foreign pathogens and abnormal host cells^[9]^. The complement system consists of multiple plasma proteins that sequentially interact with one another to mount an attack on the foreign substance. These complement proteins are widely dispersed throughout the body fluids and tissues and remain dormant until an infection or an abnormality is recognized. At that point, they are locally activated and will trigger a series of strong inflammatory events. Three different pathways can trigger the activation of the complement system: the classical pathway, alternative pathway, and lectin pathway. Though these pathways differ in response to the local stimuli that cause their activation, all of these pathways lead to the cleavage of complement protein C3 into C3a and C3b, and C5 into C5a and C5b. C3a and C5a are inflammatory mediators or anaphylatoxins, and they contribute to inflammation through stimulation of histamine release and activation of other immune cells such as macrophages, eosinophils, and neutrophils^[10]^. C3b aids in opsonization which helps with phagocytosis of pathogens, and C5b initiates the assembly of the membrane attack complex, which is an essential mechanism to eliminate bacteria resistant to phagocytosis. Though the complement system is an important arm of the immune system, insufficient stimulation and/or overstimulation can be deadly to the host and is associated with autoimmune problems, chronic inflammation, infections, and even cancer^[9]^. 

### Complement system and cancer

The complement system has been regarded as a component of the innate immune response against invading pathogens and “non-self-cells”. However, when a tumor or malignancy is identified, the levels of complement proteins in tissues and fluids appear to vary^[8]^. It has been shown that the expression of complement proteins is increased in malignant tumors^[8]^. Complement system activation in the tumor microenvironment has been shown to lead to enhanced tumorigenesis and progression^[8]^. 

It has been well established that an inflammatory process is vital for the development of tumors in humans^[11]^. It was recognized that cancers develop from “subthreshold neoplastic states'' which are caused by carcinogens that elicit a somatic change to the cells, but these altered cells can stay in normal tissues indefinitely, but when the second source of stimulation such as irritation or inflammation arises, these cells start to manifest as cancerous^[5]^. 

Through numerous experiments, it has been established that activation of the complement system is a very important component of tumor-promoting inflammation^[12]^. The inflammatory response is caused by the complement system’s anaphylatoxins which are a critical step in the progression of tumorigenesis and cancer^[12]^. It was shown that C3a or C5a, which are both anaphylatoxins, can cause inflammation in a variety of ways by releasing histamine, activating other leukocytes, and stimulating the production of inflammatory mediators like TNF-alpha, IL-6, IL-1β, and IL-1^[13]^. It has been shown that these anaphylatoxins generated during tumor-associated complement activation can reshape the tumor microenvironment and play a role in tumor cell proliferation, resistance to chemotherapy, and angiogenesis within pancreatic cancer^[14]^. C5a has been shown to upregulate angiogenic factors such as vascular endothelial growth factor (VEGF) in cancer cells which plays a huge role in the development of newly formed blood vessels allowing the tumor to grow. C3a and C5a also play a huge role in the activation of tumor-associated macrophages (TAMs). These macrophages are activated by the anaphylatoxins, just like neutrophils and other leukocytes, but TAMs play a huge role in shaping the tumor microenvironment. TAMs produce a large number of strong angiogenic and lymphangiogenic growth factors, cytokines, and proteases, all of which increase neoplastic progression^[5]^. TAMs also produce IL-10, which dampens the anti-tumor response of cytotoxic T cells. In addition, TAMs cause an increase of inflammatory cytokines, which help with the invasiveness of the cancer cells allowing them to invade more tissues and spread easily.

Bonavita et al. reported that mice deficient in complement C3 were safe against chemical carcinogenesis in mesenchymal and epithelial tissue due to reduced inflammation^[15]^. It was also shown that the long pentraxin *PTX3 *gene is a very important negative regulator of inflammation and complement activation. Mice deficient in this gene had a much higher susceptibility to chemical carcinogenesis, an increase in the number of TAM’s, and an increase of inflammatory chemokines inside the tumor^[12]^. The prolonged inflammation caused by the complement system in a tumor microenvironment increases the risk of neoplasticism transformation by causing the accumulation of cytokines, chemokines, growth factors, and reactive oxygen species, all of which can fasten the creation of a tumor-supportive microenvironment^[16]^. Further, the complement system also plays a significant role in creating cancer’s defense mechanisms, especially with drug resistance. The complement’s anaphylatoxins and downstream products like the activated macrophages have specifically been identified in upregulating specific chemoresistance signaling pathways^[13]^.

### Drug resistance pathway overview in pancreatic cancer

**Figure 1:**
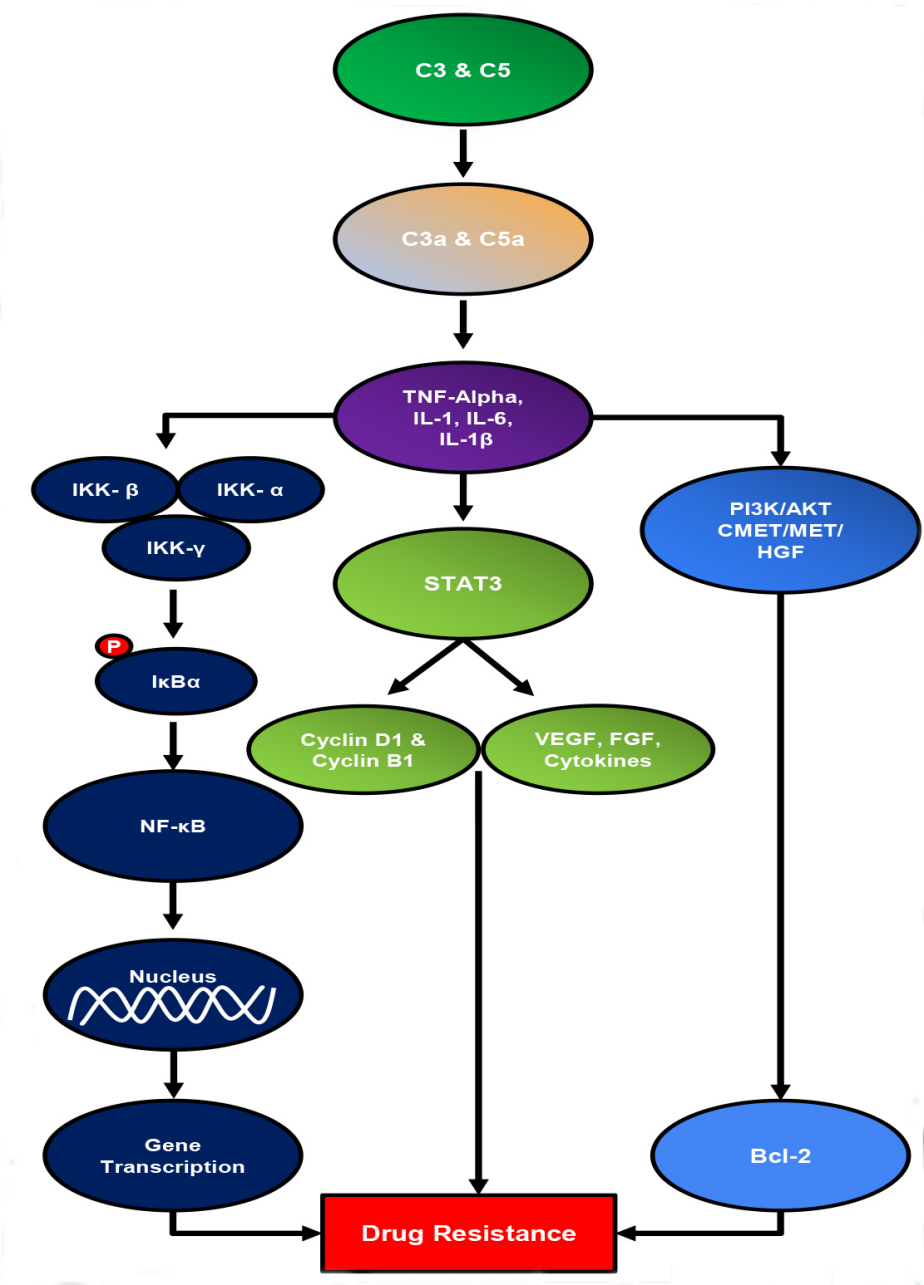
Complement system causes drug resistance through multiple signaling pathways in pancreatic cancer.

Drug resistance is one of the many reasons why the mortality rate of pancreatic cancer is so high. Through multiple experiments, it was shown that more than 165 genes are related to drug resistance^[17]^. These genes are involved with antioxidant activity, regulation of the cell cycle, signal transduction, and apoptosis. The four major chemo-resistant signaling pathways are NF-κB, C-MET, STAT3, PI3K/AKT^[18] ^[Figure 1]. 

Gemcitabine has been the standard treatment for advanced-stage pancreatic cancer since it inhibits cell proliferation and causes apoptosis of tumor cells through the activation of the adenosine monophosphate-activated protein kinase/mammalian target of rapamycin (AMPK/mTOR) pathway^[17]^. For gemcitabine to function properly, after its uptake into the cell, it must be phosphorylated by deoxycytidine kinase^[1]^. The transport, phosphorylation, and activation of gemcitabine are all governed by numerous enzymes and proteins. One protein, in particular, which is essential for the function of gemcitabine is nucleoside transporter protein such as Human Concentrative Nucleoside Transporter (hCNT1), which is responsible for taking the drug into the cell allowing for it to inhibit cellular growth and cause apoptosis^[1]^. Further, it has been demonstrated that chemo-resistant signaling pathways cause downregulation of these nucleoside transporters and inactivate deoxycytidine kinase which decreases the efficacy of gemcitabine. In addition, these signaling pathways not only inhibit the activation and transport of gemcitabine but also improve the tumor microenvironment to better protect itself from damage.

Furthermore, another major cause of resistance to gemcitabine comes from inactivity of the human equilibrative nucleoside transporter 1 (hENT-1). Gemcitabine is a pyrimidine analog, and it relies on membrane transporters for its uptake. That is why “The presence and activity of the nucleoside transporters were considered as possible important determinants of gemcitabine cytotoxicity and clinical efficacy”^[19]^. hENT-1 and hCNT-1 are the most efficient transporters, but hENT-1 is the most widely expressed in human tissues and is overexpressed in different tumor types. Without the facilitated transport function of hENT-1, the entrance of gemcitabine into the cell and its ability to cause an effect is markedly decreased. It was shown that inhibition of the facilitated, diffusion-mediated nucleoside transport by the dipyridamole derivative BIBW22BS, resulted in a 30-100-fold decrease in activity for gemcitabine in various human cancer cell lines^[20]^. Whereas, high hENT-1 levels were associated with prolonged disease-free status and overall survival in patients receiving gemcitabine adjuvant chemotherapy^[19]^.

After the entrance into the cell, gemcitabine is metabolized to active gemcitabine diphosphate and triphosphate by the enzyme deoxycytidine kinase, which leads to the inhibition of ribonucleotide reductase subunits 1 and 2, whose expression is associated with gemcitabine resistance^[21]^. In addition, gemcitabine is capable of inducing apoptosis in cancer cells primarily with a pathway involving caspase-8 and a mitochondrial-dependent caspase-9. The cascade led by caspase-8 is involved in death receptor-mediated apoptosis, whereas caspase-9 was thought to mediate chemical-induced apoptosis through the formation of an apoptosome. It was further reported that unlike inhibition of caspase 9, inhibition of caspase-8 significantly decreases apoptosis. Furthermore, stable overexpression of the caspase-9 inhibitors, caspase-9S and X-linked inhibitor of apoptosis (XIAP) in the NSCLC cell line H460 failed to suppress apoptosis induced by various chemotherapeutic drugs; however, drug-induced apoptosis was blocked in H460 transfectants that expressed known inhibitors of caspase-8^[22]^. Researchers have studied the following signaling pathways as a way to better combat the chemoresistance that they cause to improve the survival rates of patients afflicted with pancreatic cancer.

### NF-κB and complement system and its role in pancreatic cancer

Nuclear factor kappa B (NF-κB) has been shown to cause cancer progression, angiogenesis, and drug resistance in cancer, especially pancreatic cancer^[4,23]^. Ease of invasion and metastasis is one of the reasons which make the fatality rate of pancreatic cancer so high. It was shown that NF-κB was constitutively activated in about 67% of pancreatic adenocarcinoma but not in normal pancreatic tissue^[24,25]^. It has been previously reported that pancreatic cancer causes an upregulation of complement proteins, which in turn create more anaphylatoxins^[26]^. NF-κB is a transcription factor that can be activated through a classical signaling pathway or an alternative signaling pathway. The classical/canonical signaling pathway involves TNF-alpha, pro-inflammatory cytokines, and IL-1β, which will lead to the activation of the IKK-β. Then phosphorylates Iκβα, which will then cause the NF-κB dimer to be translocated into the nucleus so that it can activate gene transcription^[27]^. It was previously shown that the anaphylatoxins C3a and C5a upregulate inflammatory mediators and cytokines, and cause direct stimulation of TNF-alpha and IL-1β^[9]^. In addition, these anaphylatoxins cause more macrophages to be recruited, which have also been shown to play a huge role in inflammation^[13]^. The complement system activates NF-κB through more than just direct inflammatory mediators. The extracellular signal-regulated kinase (ERK), which is a member of the MAPK family of signaling molecules, is shown to be activated by inflammatory cytokines which are stimulated by the anaphylatoxins such as IL-6 and TNF-alpha^[28]^. Following this activation, ERK causes changes in the tumor microenvironment by producing downstream effectors such as Ribosomal S6 Kinase (RSK). RSKs have been shown to change cell proliferation and survival through the activation of NF-κB^[29]^.

Once NF-κB is activated, translocated into the nucleus, and able to affect gene expression which causes a variety of pro-tumor effects. NF-κB can cause the secretion of CXCL14 which is a chemokine that not only promotes angiogenesis and tumor growth but also plays a role in translocating NF-κB into the nucleus^[27] ^[Figure 1]. With CXCL14 and the complement system synergistically activating NF-κB, the possibility for chemoresistance rises significantly. Gastrin, a normal digestive hormone has very high levels in pancreatic cancer cells but low levels in normal tissue and is involved with metastasis through altering the expression of ABCG2 which is known as the breast cancer resistance protein through the actions of NF-κB^[4]^. However, invasion and metastasis are only a few of the many negative effects caused by the activation of this transcription factor, the most harmful being chemoresistance. Gemcitabine has been noted as the first line of defense for pancreatic cancer, but its efficacy is being decreased by the chemoresistance caused by NF-κB, which prevents improvements in survival rates and prognosis. NF-κB through a signaling pathway can cause inhibition of human concentrative nucleoside transporter 1 and 3 and human equilibrative nucleoside transporters necessary for the uptake of gemcitabine into a tumor cell^[30]^. Without the ability to properly uptake the gemcitabine, the tumor cells will continue to survive and grow, decreasing the survival rate at a steadfast rate. It was also shown that NF-κB can induce drug resistance through the upregulation of the *MDR1 *gene in cancer cells. The *MDR1 *gene encodes for an integral membrane protein, P glycoprotein, which has a specific role in drug efflux, which is the ability of the cell to regulate its internal environment and remove harmful toxins or metabolites^[31]^. This is how the cancer cells can protect themselves from chemotherapeutic treatments. It was shown that inhibiting NF-κB would downregulate this gene and the P glycoprotein which would sensitize the cells to apoptosis. NF-κB has a multitude of pro-cancerous effectors, and it has been demonstrated that the complement pathway and its downstream effectors play a role in its activation.

### Complement system and C-MET/MET/ hepatocyte growth factor pathway and its role in pancreatic cancer 

C-MET is the mesenchymal-epithelial transition factor gene encoded for membrane-bound receptor tyrosine kinase RTKs. They are expressed by epithelial cells such as the liver, pancreas, prostate, and kidney, and this receptor binds with a ligand hepatocyte growth factor (HGF). Afterwards it activates a wide range of different signaling pathways, one being chemoresistance and tumorigenesis^[32]^. There is an aberrant activation of C-MET in tumors exhibiting malignant properties. Pancreatic cancer cells are one of the most malignant neoplasms across the world, so C-MET activation would be highly expressed in this cancer^[33]^. It has been shown that C-MET expression levels are 5-7 times higher in individuals with PDAC than with normal pancreatic tissue^[34]^. Interestingly, aberrant activation of C-MET can be caused by inflammatory cytokines^[32]^. The complement anaphylatoxins can cause this aberrant activation and initiate the signaling cascade that leads to chemoresistance and other pro-tumor effects [Figure 1].

HGF-MET signaling has been shown to encourage angiogenesis in cancer by inducing VEGF expression. In addition, HGF-MET stimulation induces apoptosis protection for non-self-lung cancer cells by down-regulating apoptosis-inducing factors^[35]^. It has also been shown that over-stimulation of C-MET causes non-self-cell lung cancer resistance to an EGFR inhibitor. Further, the HGF-MET pathway supports resistance through the PI3K/AKT signaling pathway. It has been shown that C-MET deletion in pancreatic neoplasia enhanced chemosensitivity to gemcitabine. The complement systems downstream inflammatory mediators activate the C-MET pathway, which causes chemoresistance through cross-talk with other signaling pathways such as PI3K/AKT.

### Complement system and STAT3 and its role in pancreatic cancer

STAT3 play a major role in many signaling pathways and have been proven to play a role in promoting pancreatic cancer progression^[36] ^[Figure 1]. STAT3 activation is dependent on the phosphorylation of tyrosine residue, Tyr705, which then allows dimerization of STAT3 allowing it to translocate into the nucleus and affect gene transcription^[36]^. Growth factors and oncogene proteins like Src and RAS have the ability to induce the phosphorylation of the Tyrosine residue and cause STAT3 activation. However, it was shown that inflammatory cytokines such as IL-6 and TNF-alpha also have the ability to cause activation of STAT3^[37]^. It has also been reported that these inflammatory cytokines are upregulated by the anaphylatoxins which have been shown to be in higher levels within pancreatic cancer patients. The anaphylatoxins produced by the complement system have proven to play a pivotal role in inflammation and cancer progression, and its downstream effectors, like Interleukin 6, cause the constitutive activation of STAT3^[36]^. It has also been shown that C3 complement overexpression showed a clear increase in STAT3 expression in gastric cancer cell lines^[38]^.

Once the STAT3 signaling cascade has been activated, it can affect multiple aspects of cancer progression. For example, through the expression of angiogenic growth factors such as VEGF and FGF, angiogenesis of the tumor cell can be upregulated, which increases the ability to create its blood vessels and fasten the growth process. In addition, apoptosis can be inhibited through the upregulation of Cyclin D1 and Cyclin B1, which are regulatory proteins that have been implicated in tumorigenesis and the development of malignancy^[39]^. In concert with the complement system, STAT3 can upregulate inflammatory cytokines to increase inflammation and immune evasion. STAT3 can also enable the inhibitory functions of Treg cells^[37]^. Treg or T regulatory cells are responsible for maintaining peripheral tolerance and regulating the immune response. Now, if their inhibitory functions are upregulating, this in turn, causes an increase in the immunosuppressive environment in which cancer cells thrive. Finally, STAT3 plays a major role in energy metabolism for cancer cells. Cancer cells need the energy to replicate, so STAT3 upregulates glycolysis to make more ATP^[40]^. It has been shown that the energy cancer cells utilize, nearly 50%, comes from glycolysis, so the upregulation is a crucial prerequisite to tumor growth and cancer progression^[40]^. Finally, STAT3 plays an important role in chemoresistance in cancer cells. Further, STAT3 alters ATP-binding cassette membrane transporters, which are crucial to the process of drug uptake into the cell. If the drug is not able to breach the cell, then the therapeutic effect cannot be achieved^[41]^. Therefore, all the above reports suggest that STAT3 has been proven to enhance cancer progression and chemoresistance, and its phosphorylation can be initiated by the complement pathway’s anaphylatoxins.

### Complement system and PI3K/AKT and its role in pancreatic cancer

PI3K is a lipid kinase that regulates multiple cellular processes with its downstream effector, AKT. The PI3K/AKT signaling pathway has been considered a significant cause of chemoresistance in cancer therapy, inhibition of apoptosis, stimulation of cell growth, and modulation of cellular metabolism^[42] ^[Figure 1]. This signaling pathway causes multi-drug resistance through interactions with ABC transporters, NF-κB, and others.

The PI3K/AKT pathway is activated by the production of 3-phosphorylated phosphoinositide which initiates the signaling cascade. However, it was shown that C3a binding to the C3a receptor leads to transduction of intracellular signals through various G-proteins and phosphorylation of the PI3K/AKT pathway, which causes chemokine synthesis in humans. Furthermore, C3a and C5a binding to their receptors cause an influx of calcium which initiates signaling cascades such as MAPK and AKT pathways^[10,13]^. Not only does the complement pathway use the PI3K/AKT pathway to help with the formation of their chemokines, but the binding of anaphylatoxins to their respective receptors also causes the activation of this pathway. In addition, IL-6, which is released by the anaphylatoxins, is required for pancreatic cancer progression through the activation of MAPK and PI3K/AKT signaling pathway^[43]^. One of the ways to activate the PI3K/AKT pathway is through the activation of the mutated *Kras *gene synergistically acting with IL-6. *Kras *mutation is shown to be the most commonly mutated gene in pancreatic cancer and drives the formation of pancreatic intraepithelial neoplasia^[43]^. It was also shown that without IL-6, cancer progression slows even in the presence of the *Kras *mutation^[43]^. 

Once the PI3K/AKT pathway has been activated, numerous downstream effectors cause cancer progression. For example, abnormal activation of the PI3K/AKT signaling pathway contributes to the upregulation of Bcl-2 expression, which causes cell survival by inhibiting the release of cytochrome c from the mitochondria^[42]^. This type of apoptosis prevention only adds to the multi-drug resistance already previously seen. Further, another downstream effector seen in many cancers is XIAP which has played a role in chemoresistance by inhibiting autophagy-induced apoptosis. It was shown that abnormal activation of PI3K/AKT induces XIAP expression^[42]^. Especially in pancreatic cancer cells, there is a cross-talk and activation between PI3K/AKT and NF-κB which results in multidrug resistance through the stimulation of Cyclin D1 to accelerate cell progression and tumor growth leading to drug resistance. Finally, pancreatic ductal preneoplastic lesions come from the differentiation of acinar cells to ductal cells, and the process of this occurring is called acinar to ductal metaplasia (ADM). This process is accelerated by mutated *Kras *and inflammation. However, p110-alpha, which is a catalytic subunit of PI3K-α can cause ADM in the presence or absence of pancreatic inflammation^[44]^. 

The complement-induced PI3K/AKT pathway not only acts with the *Kras *mutation to fasten the cancer progression, but also with NF-κB and other mediators to promote cell survival and chemoresistance.

## CONCLUSION

The complement pathway is a part of the body’s innate immune response capable of attacking pathogens and foreign abnormalities. However, with cancer, the complement pathway does more harm than good. It allows for increased inflammation through the release of anaphylatoxins, the activation of different signaling cascades through the induction of inflammatory mediators, and finally a nourishing tumor microenvironment. The most detrimental side effect of the complement system’s activation would be the chemoresistance caused by the anaphylatoxins.

Pancreatic cancer has had one of the highest cancer-related mortality rates due to its fast metastasis and chemoresistance. The major signaling pathways discussed in this review article discussed have all been targets of therapeutic treatment in order to combat chemoresistance. Though these treatments have been shown to cause higher sensitivity to gemcitabine and other chemotherapeutic drugs, the problem has not been solved. The fact of the matter is that all of these signaling pathways are activated by different stimuli and can a cross-talk, therefore, inhibiting each one yields a futile response. However, it has been shown that targeting the complement pathway has been successful as a therapeutic treatment option for lung cancer in a few pre-clinical studies which indicate that inhibition of either C3a or C5a signaling inhibits cancer progression in lung cancer models and other malignancies^[45]^. However, even with all the current evidence showing how the complement system upregulates inflammation and mediators that enable tumorigenesis, as of 2019, no clinical trials are targeting the complement system as a therapeutic approach. It has been shown that the complement system upregulates these signaling cascades, and targeting each main pathway has proven to be ineffective. If the complement’s anaphylatoxins were targeted and the inflammatory response was removed, then there would be fewer inflammatory mediators upregulating these pathways. This could yield a more successful chemotherapeutic treatment without the obstacle of chemoresistance. 

## DECLARATIONS

### Copyright

© The Author(s) 2022.
